# Jintiange Capsules Ameliorate Osteoarthritis by Modulating Subchondral Bone Remodeling and Protecting Cartilage Against Degradation

**DOI:** 10.3389/fphar.2021.762543

**Published:** 2021-11-11

**Authors:** Chenyang Zhuang, Zixiang Wang, Weisin Chen, Hanquan Wang, Bo Tian, Hong Lin

**Affiliations:** ^1^ Department of Orthopedics, Zhongshan Hospital, Fudan University, Shanghai, China; ^2^ Department of Orthopedics, Shanghai Geriatric Medical Centre, Fudan University, Shanghai, China

**Keywords:** Jintiange capsule, osteoarthritis, subchondral bone, cartilage, osteoclast, chondrocyte

## Abstract

Osteoarthritis (OA) is the most prevalent joint disease worldwide, making it a major cause of pain and disability. Identified as a chronic and progressive disease, effective treatment at the early stages of OA has become critical to its management. Jintiange (Jtg) capsules are a traditional Chinese medicine produced from multiple organic components of various animal bones and routinely used to treat osteoporosis in China. However, the effect of Jtg on subchondral bone and cartilage degeneration in OA remains unknown. The purpose of the present study was to investigate the biomolecular role and underlying mechanisms of Jtg in OA progression. Herein, we found that Jtg inhibited receptor activator of nuclear factor-κB ligand (RANKL)-induced osteoclast formation and it functions through the NF-κB signaling pathway. Jtg also inhibited chondrocyte apoptosis via reducing the reactive oxygen species concentration in these cells. Moreover, *in vivo* evaluation revealed that Jtg significantly attenuates subchondral bone remodeling and cartilage destruction in anterior cruciate ligament transection (ACLT) mouse models. Taken together, our data demonstrate that Jtg inhibits osteoclast differentiation in subchondral bone and chondrocyte apoptosis in cartilage, supporting its potential therapeutic value for treating OA.

## Introduction

Osteoarthritis (OA), the most common chronic and degenerative joint disease globally, greatly threatens the quality of life of millions of people, especially the elderly. OA affects approximately 10% of men and 18% of women aged >60 years (Bijlsma et al., 2011; Litwic et al., 2013). Pain, joint stiffness or deformity, and even disability at the end stages are commonly reported in studies from around the world as typical symptoms of OA (Hochberg et al., 2013; Riegger and Brenner, 2020). However, existing pharmacotherapies focus on pain relief with no effective disease-modifying effects. Joint replacement surgery is routinely performed to improve joint function for end-stage OA patients, but the economic cost and complications associated with this type of intervention should not be ignored (Arden et al., 2021). Hence, there is a growing need to investigate and identify effective early-stage OA treatment schemes targeting underlying pathogenesis.

OA is primarily characterized by the degradation of the articular cartilage and the remodeling of the subchondral bone, which indicate aberrant activity in the chondrocytes and osteoclasts, respectively (Hunter and Bierma-Zeinstra, 2019). Chondrocytes, which account for 1–5% of the total cartilage volume, are unique cells specifically responsible for the physiological function and composition of the cartilage (Glyn-Jones et al., 2015). Patients with OA exhibit increased chondrocyte apoptosis, which is closely associated with the degradation of extracellular matrix components such as type II collagen and aggrecan. Increasing compelling evidence indicates that chondrocytes undergo apoptosis and induce cartilage destruction in response to various stimuli including mechanical, inflammatory, and metabolic factors and was, until recently, considered the primary pathogenic driver of OA (Lepetsos and Papavassiliou, 2016; Bolduc et al., 2019). However, experimental animal studies have shown that OA is not the inevitable result of scarification in the articular cartilage, suggesting that more specialized mechanisms are involved in OA associated cartilage degradation (Meachim, 1964; Meachim et al., 1965). There is increasing evidence that bone changes in the subchondral bone occur earlier than cartilage destruction in early-stage OA, and alterations in the subchondral bone are likely to be early mediators of OA progression (Goldring, 2009; Goldring and Goldring, 2010). The subchondral bone is a vital support of the articular cartilage, indicating that it has distinct effects on the stress dispersion and conformation of joints; these effects are significantly suppressed in OA joints. Tensile and shear stresses as well as other mechanical overload are exacerbated in subchondral bone plates with lower density and stiffness, further exposing articular cartilage to abnormal mechanical and biochemical effects. In early-stage OA, hyperactivity in the osteoclasts and increasing subchondral bone remodeling were the driving forces behind the bone loss in and thinning of the subchondral bone plate, which induced the catabolism of the articular cartilage. Recently, researchers have suggested that many factors may dysregulate homeostasis of both subchondral bone and cartilage microenvironments *via* the activation of certain signaling pathways. It has been demonstrated that aberrant activation of transforming growth factor *ß* (TGF-β) through osteoclastogenesis in subchondral bone in response to abnormal mechanical loading in onset of OA induces apoptosis of chondrocytes, thereby blocking extracellular matrix integrity in cartilage (Hu et al., 2020). This means that interventions designed to target osteoclastogenesis in subchondral bone are expected to be the most effective approaches to designing novel therapeutics for early-stage OA.

Non-steroidal anti-inflammatory drugs (NSAIDs) are the first-line treatment for OA and demonstrate significant pain-relieving effects, but these effects are accompanied by many safety concerns, including increased risks for gastrointestinal and cardiovascular adverse events. In addition, NSAIDs demonstrate clinically relevant reduction in symptoms without disease-modifying effects. It is hoped that traditional Chinese medicine-based approaches may have the potential and flexibility to help develop novel combination therapies for treating OA (Kong and Wen, 2019; Tang, 2019; Wu et al., 2019). Many theories of traditional Chinese medicine provide anecdotal evidence that bones from certain animals such as tigers, cattle and horses can inhibit bone resorption and remodeling (Ma et al., 2011; Suntornsaratoon et al., 2018). Artificial tiger bone powder, also called Jintiange (Jtg) capsules, is mixed in a pre-determined ratio with calcium, collagen, bone morphogenetic protein, bone growth factors, polypeptides, amino acids, and polysaccharides and used to treat bone-related conditions. Jtg is known for having similar medical effects to real tiger bone and has been reported to have distinct effects on osteoporosis in many experimental animal and clinical studies (Sun et al., 2019). However, the effects of Jtg on articular cartilage and subchondral bone and its underlying mechanisms remain elusive.

This study aimed to determine the effects of Jtg on receptor activator of nuclear factor-κB ligand (RANKL)-induced osteoclastogenesis and chondrocyte apoptosis, which mimic the two typical cellular dysfunctions of OA *in vitro*. Our results are expected to provide some translational value in developing a novel treatment strategy incorporating elements from traditional Chinese medicine for early-stage OA treatment.

## Materials and Methods

### Media and Reagents

Jtg (Purity > 95%) was obtained from Ginwa (Xi’an, China), and dissolved in Dimethyl sulfoxide (DMSO) (Sigma-Aldrich, Milwaukee, WI, United States). α-minimum essential medium (α-MEM) and Dulbecco’s minimum essential medium (DMEM) were purchased from Hyclone (Logan, UT, United States). Fetal bovine serum, insulin-transferrin-selenite (Livshits et al., 2010) and penicillin/streptomycin were purchased from Gibco (New York, NY, United States). Receptor activator of nuclear factor-κB ligand (RANKL) and macrophage colony stimulating factor (M-CSF) were obtained from R&D Systems (Minneapolis, MN, United States). H_2_O_2_ solution, the TUNEL apoptosis detection kit, cell counting kit-8 assay, DCFH-DA probe and RIPA lysis buffer were obtained from Beyotime Biotechnology (Shanghai, China). Antibodies against nuclear factor-kappa B (NF-κB) and mitogen-activated protein kinase (MAPK) were purchased from Cell Signaling Technology (Danvers, MA, United States). Rhodamine-conjugated phalloidin, 4′,6-diamidino-2-phenylindole (DAPI) and TRIzol reagent were obtained from Invitrogen (Carlsbad, CA, United States).

### Cell Culture

Primary bone marrow stromal cells (BMSCs) were isolated from 6-week-old, female C57BL/6 mice by flushing their femurs and tibias with α-MEM (adding 10% FBS, 1% penicillin and streptomycin). After supplementing the medium with macrophage colony stimulating factor (M-CSF, 30 ng/ml) and incubating in a humidified atmosphere of 5% CO2 at 37°C, primary bone marrow macrophages (BMMs) were obtained. The murine chondroprogenitor cell line ATDC5 purchased from the National Collection of Authenticated Cell Cultures (Shanghai, China) were cultured in DMEM (adding 10% FBS, 1% penicillin and streptomycin). ATDC5 is a well-established culture model *in vitro*, which has been commonly used for chondrogenic differentiation in the previous studies (Yao and Wang, 2013). To induce differentiation of the cells into chondrocyte-like cells, the medium containing DMEM and 5% FBS was supplemented with 1% ITS and the medium was changed every 2 days until the culture reached 80% confluence.

### Cell Viability Assay

The cytotoxic effects of Jtg on BMMs and ATDC5 cells were evaluated with a cell counting kit-8 assay (CCK-8) following the manufacturer’s instruction. BMMs were seeded in 96-well plates at a density of 1 × 10^4^ cells/well in triplicate, and co-cultured in 100 μL complete α-MEM medium with 30 ng/mL M-CSF and different concentrations of Jtg (0, 1.25, 2.5, 5, 10, 20, 40 mg/L) for 24 h. Similarly, ATDC5 cells were cultured with different concentrations of Jtg (0, 1.25, 2.5, 5, 10, 20, 40 mg/L) for 24 h. After washing with PBS for three times, 10 μL CCK-8 buffer was added to each well and incubated at 37°C for additional 2 h. Then, the absorbance was measured at a wavelength of 450 nm and quantitated as the percentage compared with the untreated control group with a microplate reader (Bio-Tech, CA, United States).

### Osteoclastogenesis and Bone Resorption Pit Assay

To further differentiate osteoclasts, the BMMs obtained as mentioned above were seeded in a 96-well plate with 6 × 10^3^ cells per well and treated with complete α-MEM medium containing 30 ng/mL M-CSF, 50 ng/ml RANKL and different concentrations of Jtg (0, 5, 10 mg/L). After changing the medium every other day for 5 days, cells were fixed with 4% paraformaldehyde for 20 min and stained for TRAP activity. TRAP-positive osteoclasts with no less than 3 nuclei were scored under a microscope. To perform the bone resorption pit assay, BMMs were seeded on the bovine bone slices in a 96-well plate and treated with α-MEM medium containing 30 ng/mL M-CSF, 50 ng/ml RANKL, and different concentrations of Jtg (0, 5, 10 mg/L). After changing the medium every other day for 5 days, the slides were washed with PBS and further treated with mechanical agitation and sonication to completely remove the cells. Bone resorption pits were observed using scanning electron microscopy (HITACHI-S520; Hitachi, Tokyo, Japan) and quantified using ImageJ software (National Institutes of Health, Bethesda, MD, United States).

### Immunofluorescence Staining of F-Actin Ring

BMMs were seeded in 35 mm glass bottom microwell dishes at the concentration of 6 × 10^3^ cells per well and cultured with α-MEM medium containing 30 ng/mL M-CSF, 50 ng/ml RANKL, with or without Jtg. After formation of mature osteoclasts, cells were fixed with 4% paraformaldehyde for 20 min and subsequently permeabilized with 0.1% Triton X-100 for 5 min. Fixed cells were stained with rhodamine-conjugated phalloidin for 1.5 h. The cells were then mounted on slides, and the nuclei were stained with 4′,6-diamidino-2-phenylindole (DAPI) for 5 min and were observed with confocal fluorescence microscopy (FV3000, Olympus, Tokyo, Japan).

### Measurement of ROS in ATDC5 Cells

ROS level was assessed with a DCFH-DA probe in ATDC5 cells, according to the manufacturer’s instructions. ATDC5 cells were cultured with or without various concentrations of Jtg (0, 5, 10 mg/L) and 200 μM H_2_O_2_ for 24 h based on the previous study (Zhou et al., 2019). After incubated with 10 μM DCFH-DA in the dark for 20 min at 37°C, the cells were washed three times with PBS. The fluorescence microscopy was then used to detect and count ROS-positive cells. Meanwhile, the cytometry analysis was further used to determine the ROS-positive cells stained by the DCFH-DA probe using a flow cytometer (BD Cytometer System, CA, United States).

### Quantitative Real-Time Polymerase Chain Reaction

Quantitative PCR was used to quantify the specific genes expression of osteoclastogenesis and ATDC5 cell apoptosis. BMMs were seeded in 6-well plates at a density of 1 × 10^5^ cells per well and cultured in complete α-MEM containing 30 ng/mL M-CSF and 50 ng/ml RANKL. The BMMs were treated with 10 mg/L Jtg for 0–5 days. ATDC5 cells were seeded in 6-well plates at a density of 1 × 10^5^ cells per well and treated with complete DMEM supplemented with 200 μM H_2_O_2_ and different concentrations of Jtg (0, 2.5, 5, 10 mg/L) for 24 h. Total RNA was extracted using TRIzol reagent. cDNA synthesis was performed with a reverse transcription reagent (Applied Biosystems, Foster, CA, United States) and followed with the transcription-PCR using real-time PCR (ABI 7500; Applied Biosystems, Foster City, CA). The mouse primer sequences of Cathepsin K (CTSK), TRAP, c-Fos, NFATc1, GAPDH, Bcl-2, Bcl-xL and Bak were listed in Table 1.

**TABLE 1:** Sequences of both the forward and reverse primers of mRNAs in RT-PCR.

Gene	Forward (5ʹ-3ʹ)	Reverse (3ʹ-5ʹ)
CTSK	CTT​CCA​ATA​CGT​GCA​GCA​GA	TCT​TCA​GGG​CTT​TCT​CGT​TC
TRAP	CTG​GAG​TGC​ACG​ATG​CCA​GCG​ACA	TCC​GTG​CTC​GGC​GAT​GGA​CCA​GA
c-Fos	CCA​GTC​AAG​AGC​ATC​AGC​AA	AAG​TAG​TGC​AGC​CCG​GAG​TA
NFATc1	CCG​TTG​CTT​CCA​GAA​AAT​AAC​A	TGT​GGG​ATG​TGA​ACT​CGG​AA
GAPDH	ACC​CAG​AAG​ACT​GTG​GAT​GG	CAC​ATT​GGG​GGT​AGG​AAC​AC
Bcl-2	ATG​CCT​TTG​TGG​AAC​TAT​ATG​GC	GGT​ATG​CAC​CCA​GAG​TGA​TGC
Bcl-xL	TGC​GTG​GAA​AGC​GTA​GAC​AA	ATT​CAG​GTA​AGT​GGC​CAT​CCA​A
Bak	CCC​AGG​ACA​CAG​AGG​AGG​TTT	GCC​TCC​TGT​TCC​TGC​TGA​TG

### Western Blot

BMMs were seeded in 6-well plates at a density of 6 × 10^5^ cells/well and cultured with 50 ng/ml RANKL in the presence or absence of 10 mg/L Jtg in complete α-MEM for 0, 5, 10, or 60 min. ATDC5 cells were seeded in 6-well plates at a density of 6 × 10^5^ cells/well and cultured with or without 10 mg/L Jtg and 200 μM H_2_O_2_ in complete DMEM. The total protein was extracted from BMMs and ATDC5 cells using RIPA lysis buffer according to the manufacturer’s instructions. The protein concentrations were quantitated and analyzed using a BCA protein assay kit (Thermo Pierce, Rockford, IL, United States). 30 μg of total protein were separated by 10% sodium dodecyl sulfate-polyacrylamide gel electrophoresis (SDS-PAGE) and transferred onto PVDF membranes (Millipore Corp, Billerica, MA). After being blocked by 5% skimmed milk, the membranes were incubated with primary antibodies at 4°C overnight. Furthermore, the membranes were incubated with horseradish peroxidase-conjugated secondary antibody diluted in the blocking buffer at room temperature for 1 h. Finally, the blots were visualized using Image-quant LAS 4000 Analyzer (GE Healthcare, Silverwater, NSW, Australia).

### P65 Nuclear Translocation Staining

BMMs were seeded in 6-well plates at a density of 6 × 10^5^ cells/well and cultured with 50 ng/ml RANKL in the presence or absence of 10 mg/L Jtg in complete α-MEM. Then the cells were fixed with 4% paraformaldehyde for 15 min, washed with 0.2% Triton X-100 in PBS for 5 min, blocked with 1% BSA in PBS, and incubated with monoclonal anti-P65 antibody followed by biotinylated goat anti-mouse IgG antibody as the secondary antibody. The cells were further stained with DAPI for 5 min and observed using a confocal fluorescence microscopy.

### Cell Apoptosis Measurement

An Annexin V-FITC/PI apoptosis kit was used to determine the rate of ATDC5 cells apoptosis. ATDC5 cells were seeded in 6-well plate and cultured with or without 200 μM H_2_O_2_ and various concentrations of Jtg for 24 h, followed by re-suspended in 500 μL binding buffer and stained with annexin V-FITC/PI staining. Then, the sample was tested and analyzed using the flow cytometer. The results are represented as the percentage of apoptotic cells.

### TUNEL Staining

To evaluate the alterations associated with ATDC5 cell apoptosis, TdT-UTP nick end labeling (TUNEL) staining was conducted using the one step TUNEL fluorescence kit according to the manufacturer’s instructions. After treated with or without 200 μM H_2_O_2_ and 5 or 10 mg/L Jtg for 24 h, the cells were permeabilized with 0.1% Triton X-100 for 2 min and stained with TUNEL mixture for 1 h according to the manufacturer’s instructions. The cells were further stained with DAPI for 5 min and observed using a confocal fluorescence microscopy. The cells with green fluorescence were defined as apoptotic cells.

### ACLT-Induced OA Mouse Model

ACLT model was established in 10-week-old C57BL/6J male mice purchased from the Animal Center of Zhongshan Hospital, Fudan University (Shanghai, China). After approved by the Animal Ethical Committee of Zhongshan Hospital, Fudan University (2019-065), the model is produced by complete transection of anterior cruciate ligament after direct visualization of it. A sham operation was conducted by only opening the joint capsule and visualizing the anterior cruciate ligament without transection. Mice were randomly divided into four groups as follows: Sham; ACLT; ACLT + Jtg (low dose); ACLT + Jtg (high dose); (*n* = 12 per group). The ACLT + Jtg group received Jtg (10 mg/kg and 30 mg/kg for low and high dose, respectively) dissolved in 0.9% normal saline (NS) by intragastric administration every other day. Mice in the sham group and ACLT group were administered an equivalent volume of NS. To prevent infection, each mouse was administered penicillin every day during the first 3 days after surgery. Six mice in each group were sacrificed 2 weeks after surgery and another 24 mice were euthanized 4 weeks post operation, and tibia and femur bones were collected for further micro-CT scanning and histological observation.

### Micro-CT Scanning

After fixed in 4% paraformaldehyde, specimens were scanned using micro-CT (QuantumGX; PerkinElmer, Waltham, MA, United States). The subchondral bone was evaluated using three-dimensional structural parameters including bone mineral density (BMD), bone volume fraction (BV/TV), trabecular pattern factor (Tb. PF), and subchondral bone plate thickness (SBP. Th), were analyzed.

### Histological Examination

Knee joint samples were all sectioned on a microtome at a thickness of 5 μm after decalcified in 10% EDTA and embedded in paraffin. Then hematoxylin and eosin (H&E), Safranin O and TRAP staining were performed. Osteoarthritis Research Society International (OARSI) scoring system was used to assess the progression of OA according to the previous study. Immunohistochemical staining of aggrecan, MMP-13, TGF-β, Caspase-3, and TUNEL were further performed and analyzed.

### Statistical Analysis

All the experiments were performed at least three times, and the data are presented as the mean ± SD. Statistical analysis was performed using one-way analysis of variance (ANOVA). A *p* < 0.05 was considered significant.

## Results

### Jtg Inhibits RANKL-Induced Osteoclastogenesis in BMMs

**FIGURE 1:**
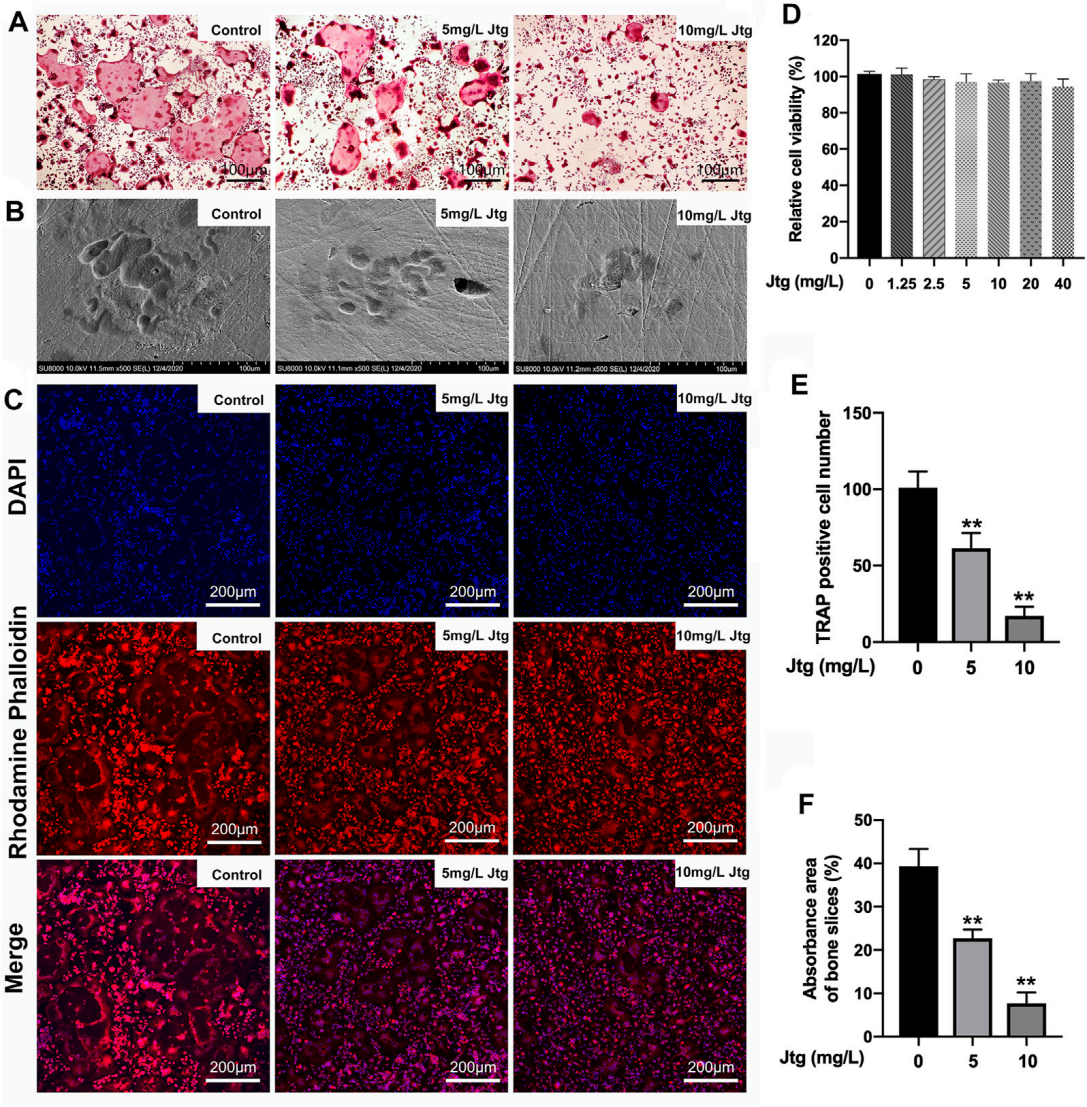
Jtg prevents receptor activator of nuclear factor-kappa B ligand (RANKL)-induced osteoclastogenesis *in vitro*. **(A)** Tartrate-resistant acid phosphatase (TRAP) staining of osteoclasts treated with different concentrations of Jtg. **(B)** Representative SEM images of bone resorption pits. **(C)** Representative images of actin rings formation were observed with confocal microscopy. **(D)** BMMs were treated with various concentrations of Jtg for 24 h and cell survival was measured using a CCK-8 assay. **(E)** The number of TRAP-positive cells (containing three or more nuclei) were determined. **(F)** The total areas of resorption pits were measured. *Compared with the control group (**p < 0.01).

The composition of Jtg was analyzed with HPLC (Supplementary Figure S1). We first evaluated the cytotoxic effects of different Jtg concentrations on BMMs using a CCK-8 assay. BMMs remained viable when treated with Jtg at concentrations ranging from 1.25 to 40 mg/L, and the BMMs were also incubated with M-CSF (30 ng/ml) and RANKL (50 ng/ml) to induce osteoclast differentiation. Notably, the Jtg treatment of these cells exhibited a dose-dependent inhibition of TRAP-positive mature osteoclasts (Figures 1A,D,E). Then we seeded BMMs on bovine cortical bone slices and stimulated them with RANKL and different Jtg concentrations (0, 5, and 10 mg/L) to assess the bone resorption capacity of these osteoclasts. Consequently, we observed a significant increase in bone resorption following RANKL stimulation, but this resorption was ameliorated in a dose-dependent manner when treated with Jtg (Figures 1B,F). In addition, evaluations of the F-actin ring, reflecting the cytoskeletal structure of the osteoclasts, revealed that this structure increased in size in response to RANKL and decreased in both size and number following treatment with Jtg (Figure 1C). These findings indicate that Jtg treatment effectively inhibits osteoclast differentiation and function *in vitro*.

### Jtg Inhibits NF-κB Signaling in RANKL Activated BMMs

**FIGURE 2:**
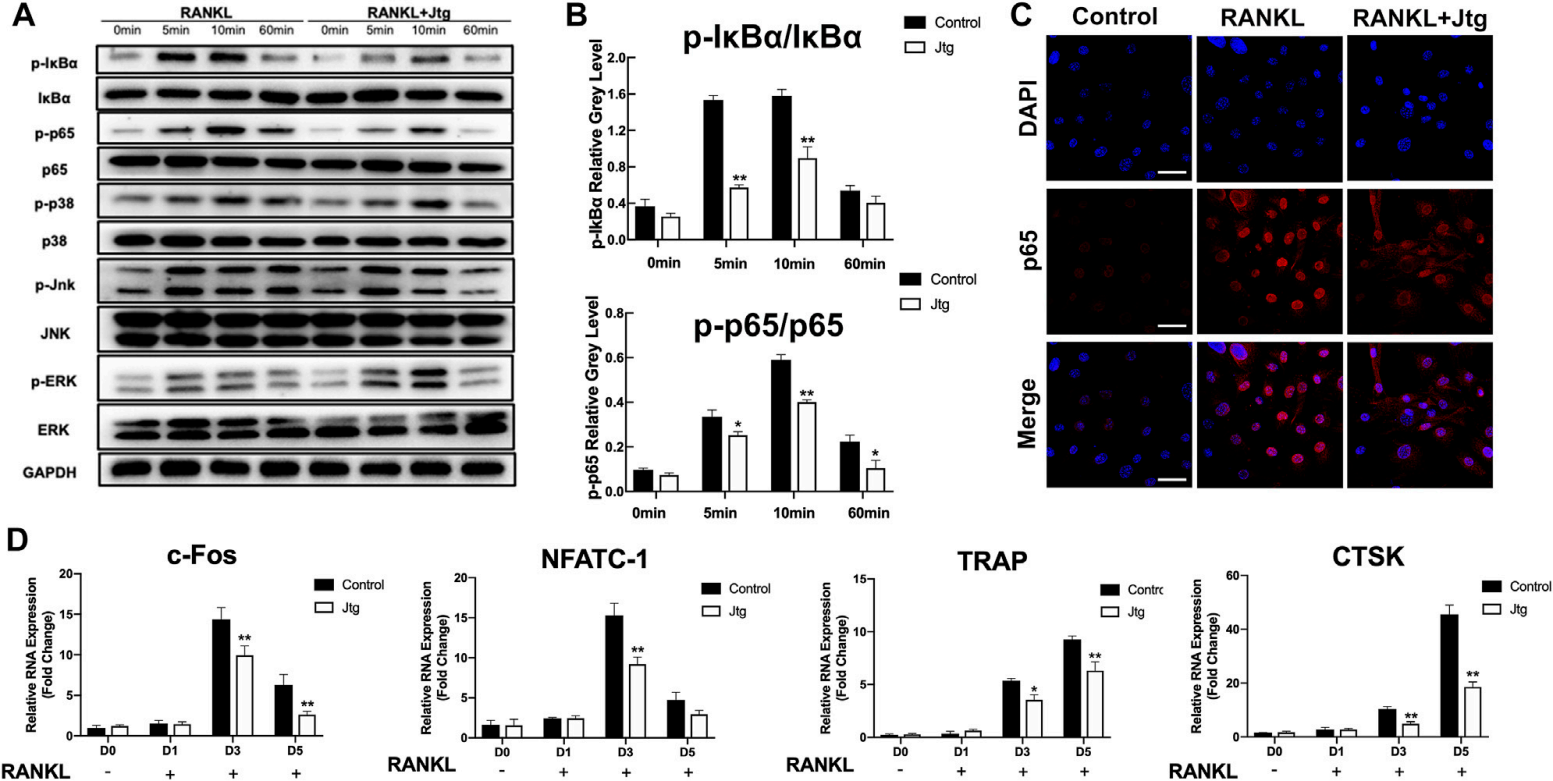
Jtg inhibits RANKL-induced osteoclast-specific gene expression and NF-κB signaling pathway without affecting MAPK pathway. **(A)** Western blot assay was performed to analyze the expression of osteoclastogenesis-related transcription factors in the NF-κB and MAPK signaling pathways including IκBα, p65, p38, ERK, and JNK. **(B)** The band intensities corresponding to p-IκBα/IκBα and p-p65/p65. **(C)** Immunofluorescence staining was performed to analyze the nuclear translocation of p65 upon RANKL stimulation in BMMs, scale bar, 50 μm. **(D)** The expression of osteoclast-specific mRNA, including c-FOS, NFATc1, TRAP, and cathepsin K (CTSK) was analyzed using RT-PCR. The mRNA-expression levels were normalized relative to the expression of GAPDH mRNA. *Compared with the control group (*p < 0.05; **p < 0.01).

We then determined the mechanisms by which Jtg treatment affects RANKL-induced osteoclastogenesis. To this end, we investigated changes in several key RANKL-signaling pathways, including the NF-κB and MAPK signaling pathways, in response to Jtg treatment using western blot. The phosphorylation of IκBa and the nuclear translocation of NF-κB (p65) are used to evaluate the activation of the NF-κB signaling pathway in BMMs. Western blot (Figures 2A,B, Supplementary Figure S2) revealed that the phosphorylation of both IκBα and p65 were significantly increased in response to RANKL. However, these effects were significantly inhibited following treatment with Jtg. Moreover, p65 nuclear translocation staining demonstrated that p65 was mostly located in the nuclear of BMMs in the control group after RANKL pretreatment while the translocation was decreased significantly in the cells cultured with Jtg (Figure 2C). These results indicated that Jtg attenuated osteoclast formation by blocking osteoclastogenesis-related NF-κB signaling. Many studies have demonstrated that the MAPK pathway is also closely associated with osteoclast differentiation. Thus, we also evaluated the interactions between Jtg and RANKL-induced MAPK signaling in an effort to further refine the underlying mechanisms of JTG treatment. However, Jtg treatment had no significant impact of the phosphorylation of p38, ERK and JNK, indicating that MAPK signaling pathway was not the mechanism mediating the effects of Jtg on osteoclastogenesis (Figure 2A, Supplementary Figure S3).

### Jtg Suppresses Osteoclast-Specific Gene Expression

We then used RT-PCR to examine the effects of Jtg on the transcription of several genes associated with osteoclastogenesis and bone resorption. BMMs were stimulated with RANKL (50 ng/ml) and then treated with 10 mg/L Jtg for 5 days and then subjected to RT-PCR. Osteoclastogenic genes, including c-FOS, NFATc1, TRAP and CTSK, were significantly upregulated by RANKL and markedly suppressed following Jtg treatment (Figure 2D), suggesting that Jtg treatment counteracts the RANKL-induced upregulation of osteoclast-specific gene expression.

### Jtg Prevents ATDC5 Cells From Undergoing ROS-Induced Apoptosis

**FIGURE 3:**
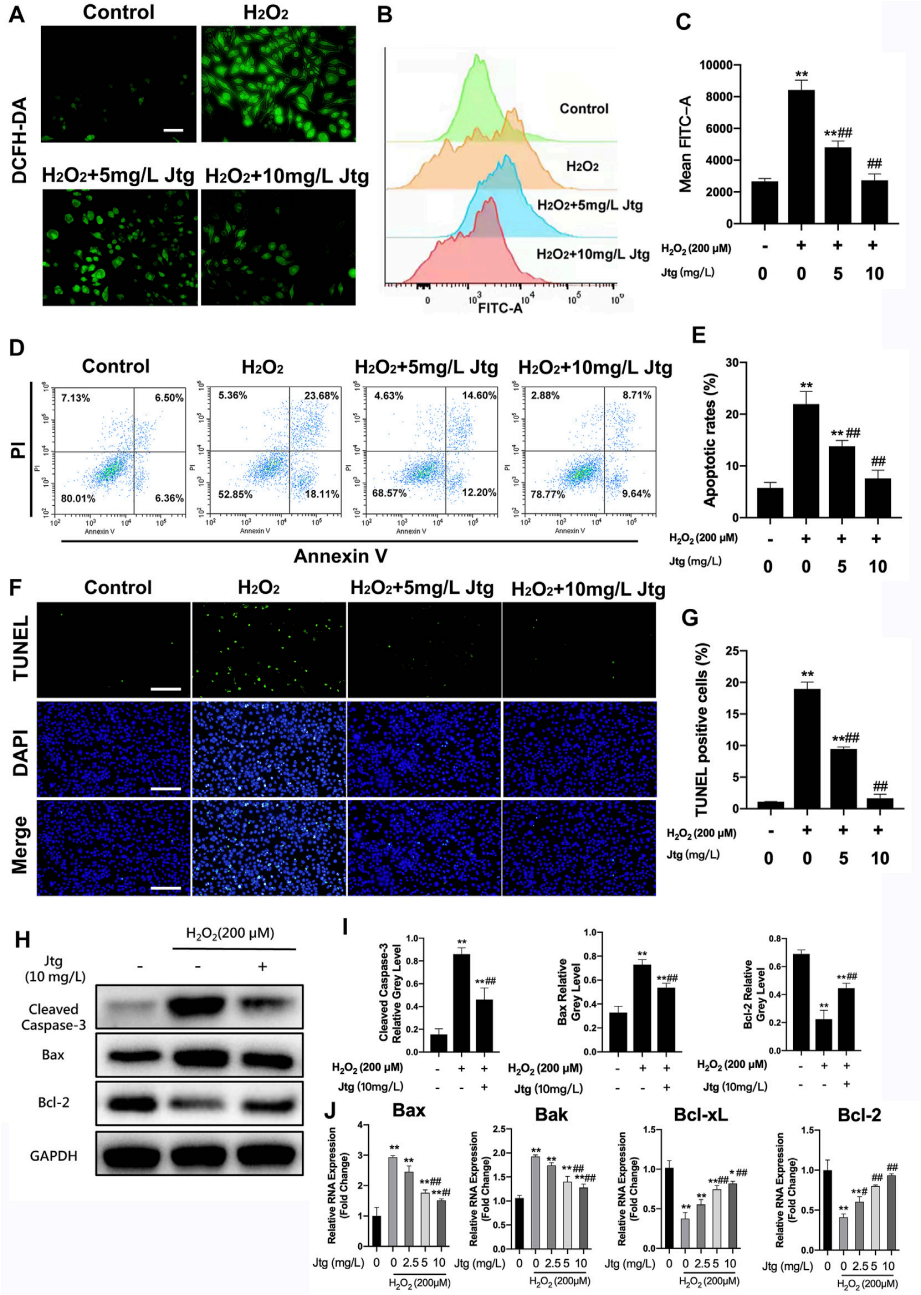
Jtg ameliorates reactive oxygen species (ROS)-induced ATDC5 cell apoptosis. **(A)** The production of intracellular ROS detected by DCFH-DA probe, scale bar, 50 μm. **(B)** ROS positive cells were detected by flow cytometry and **(C)** demonstrated as mean FITC-A level. **(D)** Cell apoptosis assessed using flow cytometry and **(E)** statistical analysis. **(F)** Detection of apoptotic cells by TUNEL and DAPI staining assay, scale bar, 200 μm and **(G)** statistical analysis. **(H, I)** Western blot assay was performed to analyze the expression of apoptosis-related proteins, including Cleaved Caspase-3, Bax and Bcl-2. **(J)** The expression of apoptosis-related mRNA, including Bcl-2 and Bcl-xL (anti-apoptotic) and Bax and Bak (pro-apoptotic), was analyzed using RT-PCR. *Compared with the control group and #compared with the H_2_O_2_ without Jtg group (*p < 0.05, ^#^ *p* < 0.05; **p < 0.01, ^##^ *p* < 0.01).

The immunofluorescence staining and flow cytometry were used to evaluate cellular ROS generation under several conditions. These evaluations revealed significant increases in ROS levels in H_2_O_2_ (200 μM) treated ATDC5 cells (Figures 3A–C), as previously described. In contrast, Jtg treatment reduced the accumulation of ROS in H_2_O_2_-treated ATDC5 cells. Flow cytometry analysis was then used to determine the apoptotic status of these cells revealing that exposure to H_2_O_2_ (200 μM) significantly increased apoptosis in ATDC5 cells Additionally, Jtg treatment could inhibit this apoptosis in a dose-dependent manner (Figures 3D,E). These results were further confirmed by TUNEL staining, which identified significantly more TUNEL positive apoptotic cells in the H_2_O_2_-treated group than in the Jtg-treated group (Figures 3F,G). These findings strongly suggest that Jtg inhibits chondrocyte apoptosis by preventing ROS accumulation.

### Jtg Blocks ROS-Induced ATDC5 Cells Apoptosis by Regulating the Expression of Key Apoptosis-Related Genes

Western blot and RT-PCR were used to determine the underlying mechanism involved in Jtg’s antiapoptotic effect in these cells. The anti-apoptotic genes Bcl-2 and Bcl-xL were downregulated, whereas the pro-apoptotic genes Bax and Bak were significantly upregulated after H_2_O_2_ stimulation. However, Jtg reversed these effects in all four apoptosis-related genes (Figure 3J). This was validated by the western blot assays that recorded similar findings for the expression of apoptosis-related proteins, including Cleaved Caspase-3, Bax and Bcl-2 (Figures 3H,I). Taken together, these results suggest that Jtg treatment alleviates ROS-induced ATDC5 apoptosis via its regulation of the expression of various apoptosis-related genes.

### Jtg Sustains Coupled Subchondral Bone Remodeling *in vivo*

**FIGURE 4:**
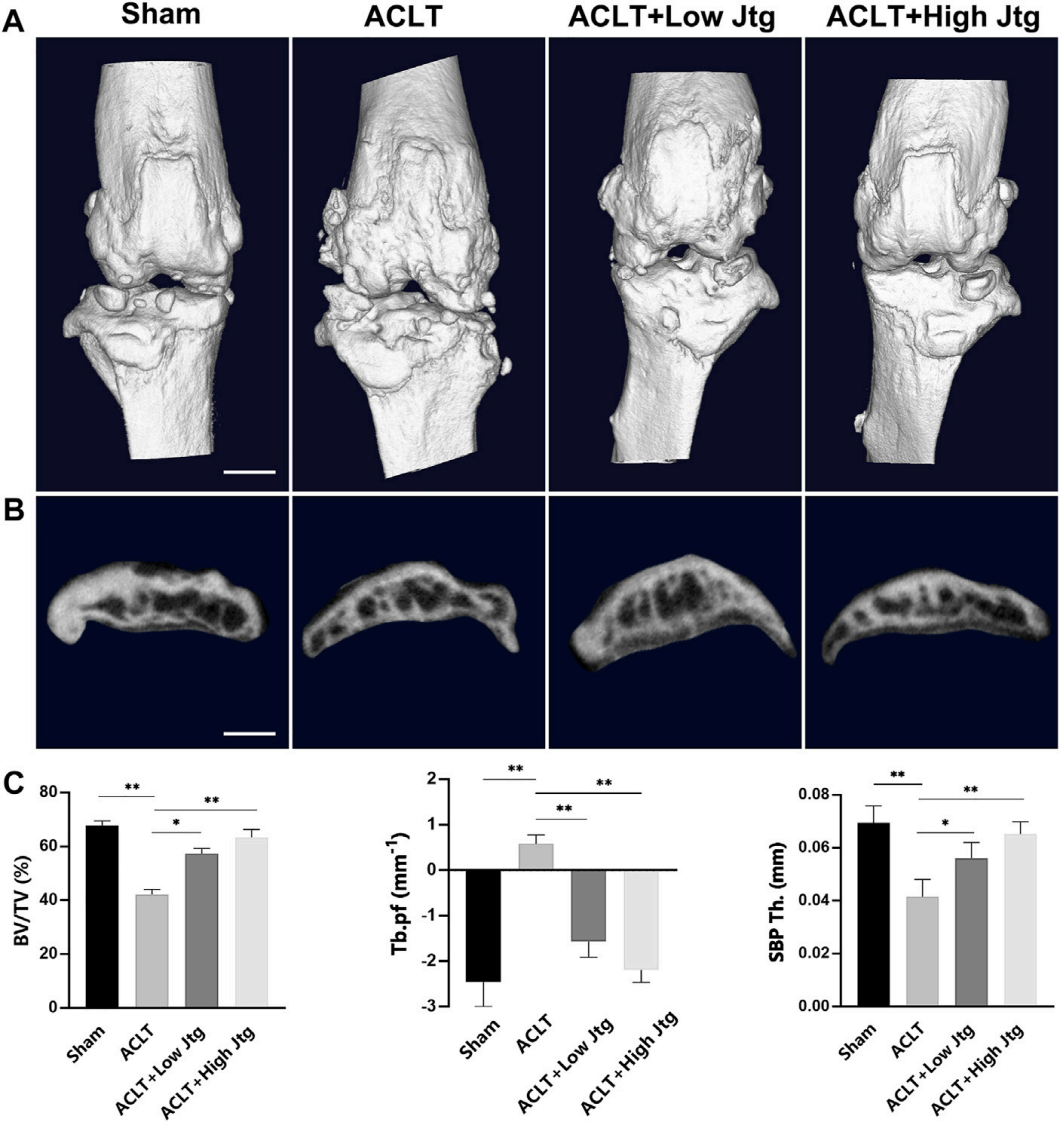
Jtg relieves abnormal subchondral bone remodeling in a mouse anterior cruciate ligament transection (ACLT) model *in vivo*. **(A)** 3D micro-CT images of frontal views of the knee joints at 4 weeks after the sham operation or the ACLT operation. **(B)** 3D micro-CT images of sagittal views of subchondral bone medial compartment after sham operation or ACLT surgery, scale bar, 1 mm. **(C)** Quantitative micro-CT analysis of tibial subchondral bone of bone volume fraction (BV/TV), trabecular pattern factor (Tb.pf), and subchondral bone plate thickness (SBP Th). *Compared with the ACLT group (*p < 0.05; **p < 0.01).

Given the positive outcomes in the *in vitro* assays we then explored the effects of Jtg on OA progression *in vivo* using an ACLT mouse model, one of the most common OA animal models. Micro-CT showed that ACLT injury induces significant subchondral bone resorption and osteophyte formation (Figures 4A,B). Both BV/TV and SBP Th. were significantly reduced in the ACLT group when compared with the Sham group, indicating the distinct influence of ACLT on subchondral bone mineralization and resorption. These animals also showed higher Tb. PF. Nevertheless, Jtg treatment restored these parameters and sustained coupled bone remodeling in a dose-dependent manner (Figure 4C).

### Jtg Inhibits Early-Stage Osteoclastogenesis and Articular Cartilage Degeneration *in vivo*

**FIGURE 5:**
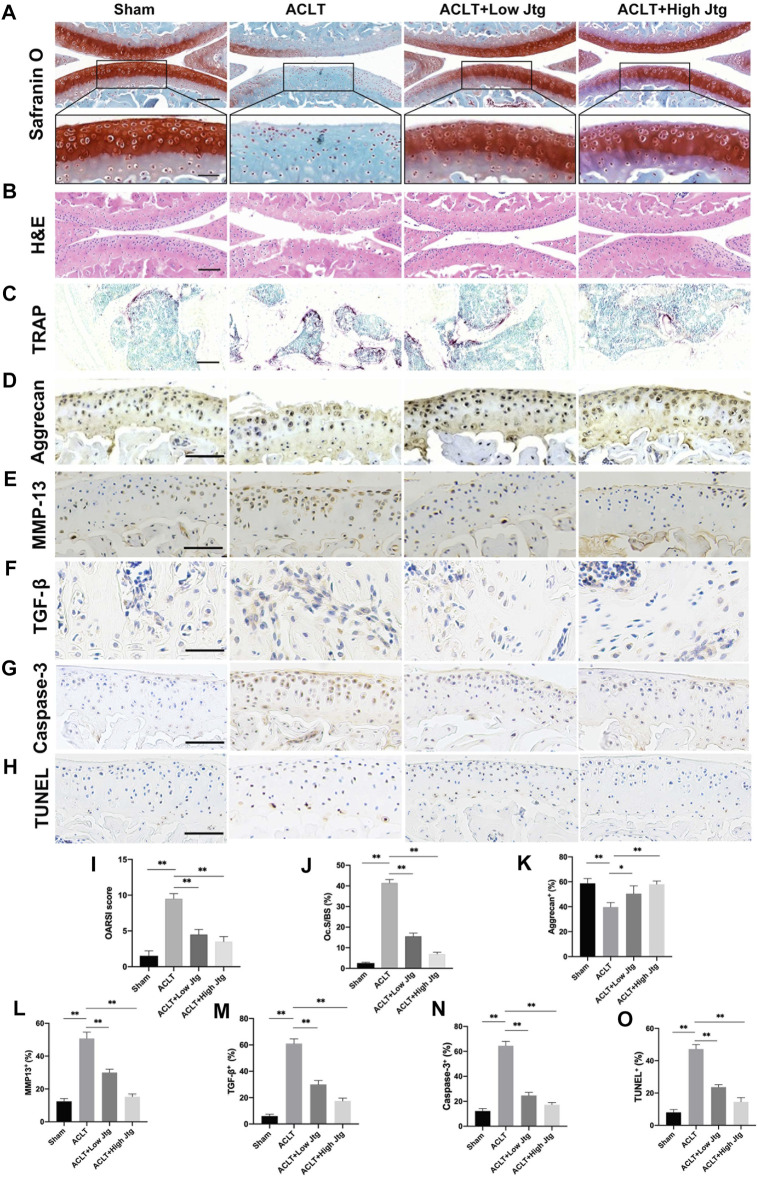
Histological analysis of Jtg treatment in a mouse anterior cruciate ligament transection (ACLT) model *in vivo*. **(A)** Safranin O staining, scale bar, 200 μm (top panels); 100 μm (bottom panels). **(B)** HE staining, scale bar, 200 μm. **(C)** Tartrate-resistant acid phosphatase (TRAP) staining, scale bar, 200 μm. **(D–H)** Immunohistochemical staining of aggrecan, MMP-13, TGF-β, Caspase-3 and TUNEL expression, scale bar, 100 μm. **(I)** Osteoarthritis Research Society International (OARSI) score indicate cartilage degeneration at 4 weeks after the ACLT operation. **(J)** Quantitative analysis of osteoclast surface per millimeter bone perimeter (Oc.s/BS) indicate subchondral bone remodeling at 2 weeks after the ACLT operation. **(K–O)** The quantitative analysis of positive cells of aggrecan, MMP-13, TGF-β, Caspase-3, and TUNEL in each group. *Compared with the ACLT group (*p < 0.05; **p < 0.01).

Safranin O/Fast Green and H&E staining were combined with the OARSI score to evaluate ACLT induced cartilage degeneration in our model. ACLT surgery significantly reduced proteoglycan and destroyed articular cartilage over a 4-week period when compared to the sham treatment control (Figures 5A,B,F). Moreover, these animals experienced early hyperactivity of the osteoclasts as shown by increased TRAP staining in the ACLT group at 2 weeks post-operation when no significant deterioration in the cartilage could be detected (Figures 5C,G, Supplementary Figure S4). However, the severity of the OA in ACLT mice was relieved following treatment with Jtg, which significantly reduced both the number of lesions and the OARSI score. We also evaluated the degree of proteoglycan and matrix degradation in these animals using immunohistochemistry against both Aggrecan and MMP-13 (Figures 5D,E). These evaluations revealed that Jtg treatment reduced the level of matrix-degrading collagenases, including MMP-13, in these samples while markedly increasing the level of Aggrecan (Figures 5H,I). Moreover, we observed highly expressed TGF-β through osteoclastogenesis in subchondral bone in ACLT mice was decreased by Jtg (Figures 5F,M). Caspase-3 and TUNEL staining demonstrated that Jtg mitigated chondrocyte apoptosis in degenerated cartilage in ACLT mice (Figures 5G,H,N,O). These findings indicate that Jtg relieves cartilage degradation and even early-stage OA while inhibiting subchondral bone resorption.

## Discussion

Traditional Chinese medicine is widely used to treat many chronic orthopedic diseases including osteoporosis, rheumatic arthritis, and OA, and it is known for its higher effectiveness and fewer side effects (Zhang et al., 2010; Lin et al., 2017; Wang et al., 2020). Jtg, a traditional medicine composed of various animal bones and organic compounds such as collagen and polysaccharides, has demonstrated outstanding efficacy in treating osteoporosis in various animal and clinical trials and is recommended for the treatment of osteoporosis in China. In addition, increasing evidence suggests that Jtg therapies may reduce bone loss and accelerate bone formation (Sun et al., 2019; Zhao et al., 2019; Zhao et al., 2021). When we combined this with the basic findings of several previous studies, we hypothesized that Jtg might also play a critical role in the treatment of another chronic orthopedic disease, OA.

Although originally thought to be restricted to the cartilage, OA is now recognized as a heterogeneous disease that affects various structures in the joint using a wide range of underlying mechanisms (Loeser et al., 2016). Osteochondral junctions play a pivotal role in the loadbearing and metabolism of the joint. As the important structure within the osteochondral junction, the subchondral bone undergoes extensive remodeling and changes all of which have been detected in early-stage OA prior to cartilage degradation by hybrid SPECT-CT, indicating its significant role in both the onset and progression of OA (Hügle and Geurts, 2017). Moreover, MRI revealed that more bone marrow lesions occur in the osteochondral junctions of OA patients which is likely a result of the increased remodeling of the subchondral bone in these joints and acts as a predictor of cartilage degeneration (Maas et al., 2015). Furthermore, accumulating evidence have shown that subchondral bone mainly undergoes a spatiotemporal uncoupled remodeling process which is notably characterized by macrophage infiltration and osteoclast activation in early-stage OA and concomitant increased osteoblast activity leading to spatial remineralization and osteosclerosis in end-stage disease (Yu et al., 2016; Jiang et al., 2021). In this study, we also attached great importance to the effects of Jtg on osteoclasts in early-stage OA. Given the close physical association between overlying cartilage and subchondral bone, the osteoclast-chondrocyte crosstalk in bone-cartilage microenvironment is a promising therapeutic target to restrain OA. Increasing evidence indicates that TGF-β plays a crucial role in osteoclast-chondrocyte crosstalk in onset of OA. Zhang et al. found that overexpression of TGF-β from osteoclasts in subchondral bone in response to altered mechanical loadings induced chondrocyte apoptosis and cartilage degeneration (Zhang et al., 2018). Moreover, Zhen et al. demonstrated that knockout of the TGF–β receptor and inhibition of TGF-β activity relieved osteoarthritis in ACLT mice (Zhen et al., 2013). Consistently, we observed aberrant TGF-β level was decreased by Jtg treatment in the subchondral bone in ACLT mice and alleviated chondrocyte apoptosis significantly, suggesting the pivotal role of Jtg in the regulation of osteoclast-chondrocyte crosstalk in OA.

Moreover, we also detected aberrant subchondral bone remodeling including decreased BV/TV and SBP Th, and increased Tb, PF, and TRAP-positive osteoclasts in early-stage OA which occurs before cartilage degradation, cementing the importance of subchondral bone remodeling in early-stage OA. Osteoclast hyperactivity is considered as the major hallmark of bone resorption. Specific interactions between RANKL and its receptor RANK play a crucial role in osteoclast formation and activation (Boyce and Xing, 2008). Meanwhile, downstream signaling transcription factors, such as NF-κB and MAPKs (p38, ERK, and JNK) are activated to further initiate osteoclastogenesis and bone resorption. Thus, any agent capable of blocking the expression of osteoclast-associated genes and signaling pathways or inhibiting RANKL/RANK interactions is likely to exert significant therapeutic effects on OA. Here, we found that Jtg suppressed RANKL-induced osteoclastogenesis in a dose-dependent manner and blocked the NF-κB signaling pathway by downregulating osteoclast-specific gene expression. This suggests that Jtg alleviates subchondral bone remodeling through the inhibition of osteoclast differentiation and activation.

As the most important constituent of cartilage, extracellular matrix components provide the tensile strength and compressive resilience facilitating the basic functionality of the joint (Rahmati et al., 2017). Increasing evidence suggests that extracellular matrix components including type II collagen and aggrecan are significantly reduced in the cartilage of OA patients. Chondrocytes, the only cells that populate avascular extracellular matrix components are responsible for this change in OA cartilage (Riegger and Brenner, 2020). As chemical byproducts of the mitochondrial electron transport chain, ROS are known to act as primary intermediates for many signaling pathways including cellular apoptosis (Zhang et al., 2016). A low-oxygen-tension environment helps to maintain the collagen turnover rate mediated by chondrocytes under physiological conditions. Oxidant-induced chondrocyte apoptosis, which is induced in response to excessive ROS generation, is known to be a crucial event in OA (Arra et al., 2020). Our findings suggest that Jtg treatment inhibits H_2_O_2_-induced chondrocyte apoptosis *in vitro*. We also showed that Jtg treatment reduced the expression level of matrix-degrading enzymes and increased the expression level of matrix proteins. Furthermore, both the Safranin O/Fast Green staining and H&E staining revealed similar outcomes *in vivo*. Thus, we also show that Jtg treatment can protect cartilage against degeneration through its inhibition of chondrocyte apoptosis.

There are several potential limitations in our study. First, the primary cells used in this study were obtained from mice instead of humans, so it is necessary to use human cells in future studies to make our results more reliable. And we just studied the potential effects of Jtg on the NF-κB and MAPK pathway, we should also detect other mediators and signaling pathways involved in the pharmacologic mechanism of Jtg in future studies.

In summary, this study demonstrates, for the first time, that Jtg treatment effectively inhibits osteoclast differentiation and activation by inhibiting NF-κB signaling cascades and ROS-induced chondrocyte apoptosis. These dual effects eventually ameliorate the subchondral bone remodeling and cartilage degeneration associated with OA in ACTL mouse models. Taken together, these results suggest that Jtg is a potential alternative therapeutic agent for attenuating early-stage OA.

## Data Availability

The original contributions presented in the study are included in the article/Supplementary Material, further inquiries can be directed to the corresponding authors.

## References

[B1] Arden N. K. Perry T. A. Bannuru R. R. Bruyère O. Cooper C. Haugen I. K. (2021). Non-surgical Management of Knee Osteoarthritis: Comparison of ESCEO and OARSI 2019 Guidelines. Nat. Rev. Rheumatol. 17 (1), 59–66. 10.1038/s41584-020-00523-9 33116279

[B2] Arra M. Swarnkar G. Ke K. Otero J. E. Ying J. Duan X. (2020). LDHA-mediated ROS Generation in Chondrocytes Is a Potential Therapeutic Target for Osteoarthritis. Nat. Commun. 11 (1), 3427. 10.1038/s41467-020-17242-0 32647171 PMC7347613

[B3] Bijlsma J. W. Berenbaum F. Lafeber F. P. (2011). Osteoarthritis: an Update with Relevance for Clinical Practice. Lancet 377 (9783), 2115–2126. 10.1016/s0140-6736(11)60243-2 21684382

[B4] Bolduc J. A. Collins J. A. Loeser R. F. (2019). Reactive Oxygen Species, Aging and Articular Cartilage Homeostasis. Free Radic. Biol. Med. 132, 73–82. 10.1016/j.freeradbiomed.2018.08.038 30176344 PMC6342625

[B5] Boyce B. F. Xing L. (2008). Functions of RANKL/RANK/OPG in Bone Modeling and Remodeling. Arch. Biochem. Biophys. 473 (2), 139–146. 10.1016/j.abb.2008.03.018 18395508 PMC2413418

[B6] Glyn-Jones S. Palmer A. J. Agricola R. Price A. J. Vincent T. L. Weinans H. (2015). Osteoarthritis. Lancet 386 (9991), 376–387. 10.1016/s0140-6736(14)60802-3 25748615

[B7] Goldring M. B. Goldring S. R. (2010). Articular Cartilage and Subchondral Bone in the Pathogenesis of Osteoarthritis. Ann. N. Y Acad. Sci. 1192, 230–237. 10.1111/j.1749-6632.2009.05240.x 20392241

[B8] Goldring S. R. (2009). Role of Bone in Osteoarthritis Pathogenesis. Med. Clin. North. Am. 93 (1), 25–35,xv. 10.1016/j.mcna.2008.09.006 19059019

[B9] Hochberg M. C. Yerges-Armstrong L. Yau M. Mitchell B. D. (2013). Genetic Epidemiology of Osteoarthritis: Recent Developments and Future Directions. Curr. Opin. Rheumatol. 25 (2), 192–197. 10.1097/BOR.0b013e32835cfb8e 23249833 PMC3771580

[B10] Hu W. Chen Y. Dou C. Dong S. (2020). Microenvironment in Subchondral Bone: Predominant Regulator for the Treatment of Osteoarthritis. Ann. Rheum. Dis. 80 (4), 413–422. 10.1136/annrheumdis-2020-218089 PMC7958096 33158879

[B11] Hügle T. Geurts J. (2017). What Drives Osteoarthritis?-Synovial versus Subchondral Bone Pathology. Rheumatology (Oxford) 56 (9), 1461–1471. 10.1093/rheumatology/kew389 28003493

[B12] Hunter D. J. Bierma-Zeinstra S. (2019). Osteoarthritis. Lancet 393 (10182), 1745–1759. 10.1016/s0140-6736(19)30417-9 31034380

[B13] Jiang A. Xu P. Sun S. Zhao Z. Tan Q. Li W. (2021). Cellular Alterations and Crosstalk in the Osteochondral Joint in Osteoarthritis and Promising Therapeutic Strategies. Connect. Tissue Res. 17, 1–11. 10.1080/03008207.2020.1870969 33397157

[B14] Kong X. Y. Wen C. P. (2019). On Research Progress of Western and Chinese Medicine Treatment on Pre-rheumatoid Arthritis. Chin. J. Integr. Med. 25 (9), 643–647. 10.1007/s11655-019-3223-3 31650484

[B15] Lepetsos P. Papavassiliou A. G. (2016). ROS/oxidative Stress Signaling in Osteoarthritis. Biochim. Biophys. Acta 1862 (4), 576–591. 10.1016/j.bbadis.2016.01.003 26769361

[B16] Lin J. Zhu J. Wang Y. Zhang N. Gober H. J. Qiu X. (2017). Chinese Single Herbs and Active Ingredients for Postmenopausal Osteoporosis: From Preclinical Evidence to Action Mechanism. Biosci. Trends 11 (5), 496–506. 10.5582/bst.2017.01216 29151553

[B17] Litwic A. Edwards M. H. Dennison E. M. Cooper C. (2013). Epidemiology and burden of Osteoarthritis. Br. Med. Bull. 105, 185–199. 10.1093/bmb/lds038 23337796 PMC3690438

[B18] Livshits G. Ermakov S. Popham M. Macgregor A. J. Sambrook P. N. Spector T. D. (2010). Evidence that Bone mineral Density Plays a Role in Degenerative Disc Disease: the UK Twin Spine Study. Ann. Rheum. Dis. 69 (12), 2102–2106. 10.1136/ard.2010.131441 20570838 PMC3002767

[B19] Loeser R. F. Collins J. A. Diekman B. O. (2016). Ageing and the Pathogenesis of Osteoarthritis. Nat. Rev. Rheumatol. 12 (7), 412–420. 10.1038/nrrheum.2016.65 27192932 PMC4938009

[B20] Ma D. Zhu W. Liang X. Long Z. Wang N. Zhang W. (2011). Effects of Enzymatic Bone Powder on Calcium Absorption and Bone Density in Rats. Wei Sheng Yan Jiu 40 (4), 492–494. 10.19813/j.cnki.weishengyanjiu.2011.04.024 21861357

[B21] Maas O. Joseph G. B. Sommer G. Wild D. Kretzschmar M. (2015). Association between Cartilage Degeneration and Subchondral Bone Remodeling in Patients with Knee Osteoarthritis Comparing MRI and (99m)Tc-DPD-SPECT/CT. Osteoarthritis Cartilage 23 (10), 1713–1720. 10.1016/j.joca.2015.05.014 26028141

[B22] Meachim G. Ghadially F. N. Collins D. H. (1965). Regressive Changes in the Superficial Layer of Human Articular Cartilage. Ann. Rheum. Dis. 24 (1), 23–30. 10.1136/ard.24.1.23 14261075 PMC1030910

[B23] Meachim G. (1964). Sulphate Metabolism of Articular Cartilage after Surgical Interference with the Joint. Ann. Rheum. Dis. 23 (5), 372–380. 10.1136/ard.23.5.372 14206215 PMC1010376

[B24] Rahmati M. Nalesso G. Mobasheri A. Mozafari M. (2017). Aging and Osteoarthritis: Central Role of the Extracellular Matrix. Ageing Res. Rev. 40, 20–30. 10.1016/j.arr.2017.07.004 28774716

[B25] Riegger J. Brenner R. E. (2020). Pathomechanisms of Posttraumatic Osteoarthritis: Chondrocyte Behavior and Fate in a Precarious Environment. Int. J. Mol. Sci. 21 (5), 1560. 10.3390/ijms21051560 PMC7084733 32106481

[B26] Sun J. Yang X. G. Hu Y. C. (2019). Efficacy of Jintiange Capsules in the Treatment of Osteoporosis: A Network Meta-Analysis. Orthop. Surg. 11 (2), 176–186. 10.1111/os.12439 30854796 PMC6594523

[B27] Suntornsaratoon P. Charoenphandhu N. Krishnamra N. (2018). Fortified Tuna Bone Powder Supplementation Increases Bone mineral Density of Lactating Rats and Their Offspring. J. Sci. Food Agric. 98 (5), 2027–2034. 10.1002/jsfa.8688 28940514

[B28] Tang C. H. (2019). Research of Pathogenesis and Novel Therapeutics in Arthritis. Int. J. Mol. Sci. 20 (7), 1646. 10.3390/ijms20071646 PMC6479975 30987068

[B29] Wang M. Liu L. Zhang C. S. Liao Z. Jing X. Fishers M. (2020). Mechanism of Traditional Chinese Medicine in Treating Knee Osteoarthritis. J. Pain Res. 13, 1421–1429. 10.2147/jpr.S247827 32606908 PMC7304682

[B30] Wu Y. R. Kuang G. Y. Lu F. G. Wang H. X. Lu M. Zhou Q. (2019). Pathological Relationship between Intestinal Flora and Osteoarthritis and Intervention Mechanism of Chinese Medicine. Chin. J. Integr. Med. 25 (9), 716–720. 10.1007/s11655-019-3224-2 31650488

[B31] Yao Y. Wang Y. (2013). ATDC5: an Excellent *In Vitro* Model Cell Line for Skeletal Development. J. Cel Biochem. 114 (6), 1223–1229. 10.1002/jcb.24467 23192741

[B32] Yu D. Xu J. Liu F. Wang X. Mao Y. Zhu Z. (2016). Subchondral Bone Changes and the Impacts on Joint Pain and Articular Cartilage Degeneration in Osteoarthritis. Clin. Exp. Rheumatol. 34 (5), 929–934. 27606839

[B33] Zhang P. Li J. Han Y. Yu X. W. Qin L. (2010). Traditional Chinese Medicine in the Treatment of Rheumatoid Arthritis: a General Review. Rheumatol. Int. 30 (6), 713–718. 10.1007/s00296-010-1370-0 20204371

[B34] Zhang J. Wang X. Vikash V. Ye Q. Wu D. Liu Y. (2016). ROS and ROS-Mediated Cellular Signaling. Oxid Med. Cel Longev 2016, 4350965. 10.1155/2016/4350965 PMC4779832 26998193

[B35] Zhang R. K. Li G. W. Zeng C. Lin C. X. Huang L. S. Huang G. X. (2018). Mechanical Stress Contributes to Osteoarthritis Development through the Activation of Transforming Growth Factor Beta 1 (TGF-β1). Bone Jt. Res. 7 (11), 587–594. 10.1302/2046-3758.711.BJR-2018-0057.R1 PMC6269596 30581556

[B36] Zhao Y. R. Wei X. Jiang J. J. Zhang Y. L. Wang S. Q. Xie Y. M. (2019). Systemic Review of Jintiange Capsules in Treatment of Postmenopausal Osteoporosis. Zhongguo Zhong Yao Za Zhi 44 (1), 186–192. 10.19540/j.cnki.cjcmm.20180709.007 30868831

[B37] Zhao J. Zeng L. Wu M. Huang H. Liang G. Yang W. (2021). Efficacy of Chinese Patent Medicine for Primary Osteoporosis: A Network Meta-Analysis. Complement. Ther. Clin. Pract. 44, 101419. 10.1016/j.ctcp.2021.101419 34049211

[B38] Zhen G. Wen C. Jia X. Li Y. Crane J. L. Mears S. C. (2013). Inhibition of TGF-β Signaling in Mesenchymal Stem Cells of Subchondral Bone Attenuates Osteoarthritis. Nat. Med. 19 (6), 704–712. 10.1038/nm.3143 23685840 PMC3676689

[B39] Zhou F. Mei J. Yuan K. Han X. Qiao H. Tang T. (2019). Isorhamnetin Attenuates Osteoarthritis by Inhibiting Osteoclastogenesis and Protecting Chondrocytes through Modulating Reactive Oxygen Species Homeostasis. J. Cel Mol Med. 23 (6), 4395–4407. 10.1111/jcmm.14333 PMC6533508 30983153

