# Supplementary material for: Jintiange Capsules Ameliorate Osteoarthritis by Modulating Subchondral Bone Remodeling and Protecting Cartilage Against Degradation
Source: Front Pharmacol. 2021 Nov 11;12:762543. doi: 10.3389/fphar.2021.762543 (PMC8631927; doi:10.3389/fphar.2021.762543)
Supplement: Supplementary file 1 [file DataSheet1.DOCX]

Supplementary Material


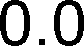

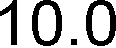

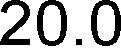

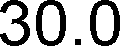

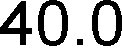

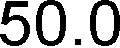

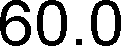

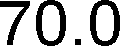

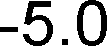

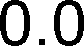

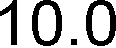

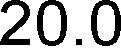

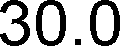

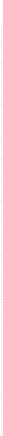

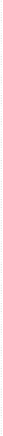

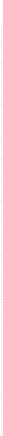

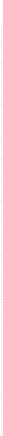

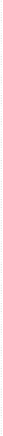

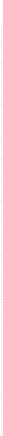

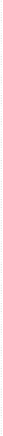

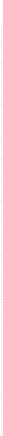

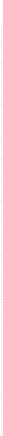

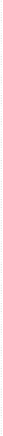

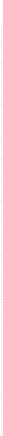

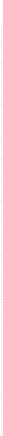

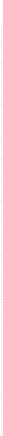

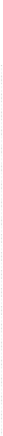

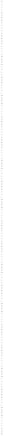

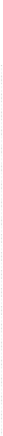

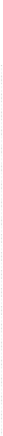

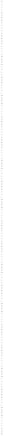

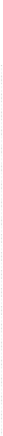

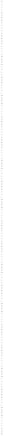

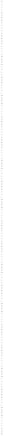

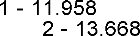

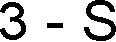

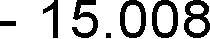

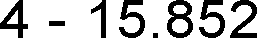

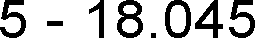

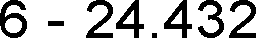

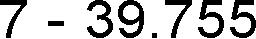


A


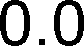

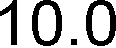

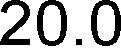

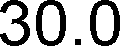

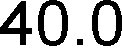

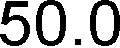

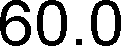

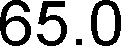

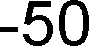

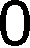

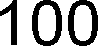

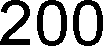

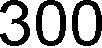

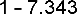

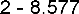

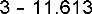

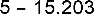

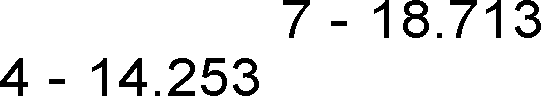

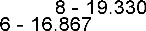

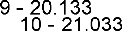

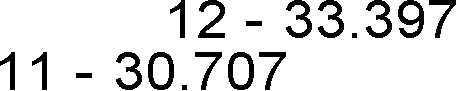

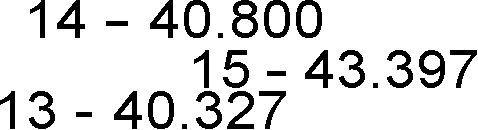

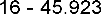


**B**

Supplementary Figure 1. (A) HPLC chromatograms of amino acids of Jtg with 16 different characteristic peaks. 1. Aspartic acid, 2. Glutamic acid, 3. Hydroxyproline, 4. Serine, 5. Glycine, 6. Histidine, 7. Arginine, 8. Threonine, 9. Alanine, 10. Proline, 11. Tyrosine, 12. Valine, 13. Leucine, 14. Isoleucine, 15. Phenylalanine, 16. Lysine. (B) HPLC chromatograms of polypeptides of Jtg with 7 different characteristic peaks.


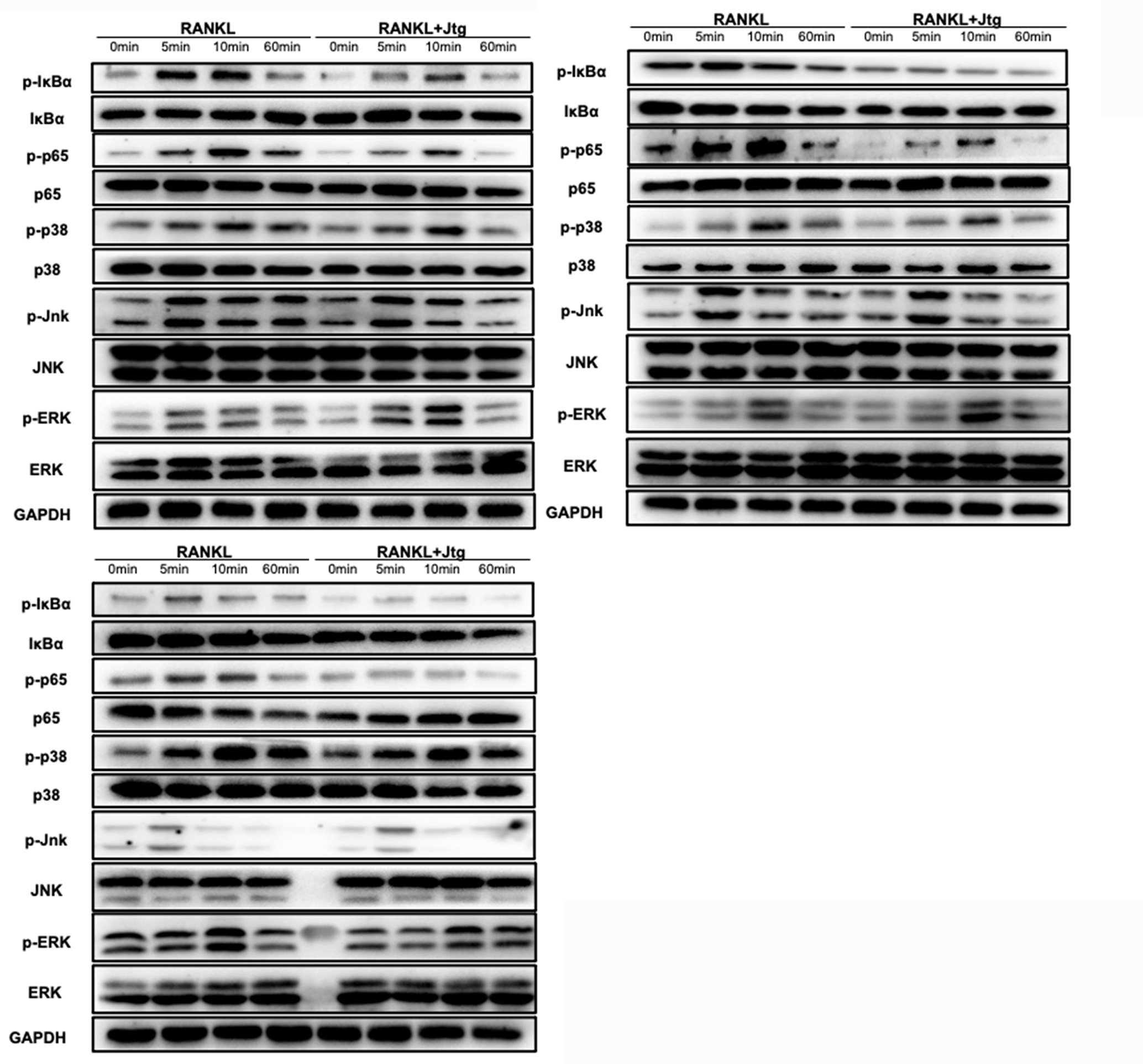


**Supplementary Figure 2.** Uncropped images of the original western blots in triplicate.

.


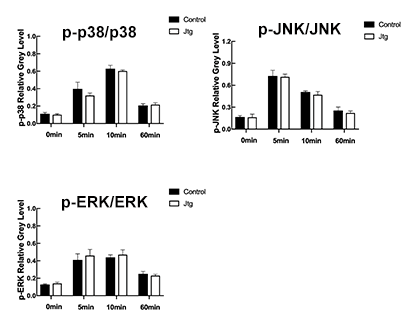


Supplementary Figure 3. Jtg shows no effect on relative proteins of MAPK pathway compared with

the control group. The band intensities corresponding to p-p38/p38, p-JNK/JNK and p-ERK/ERK were

quantified.

**
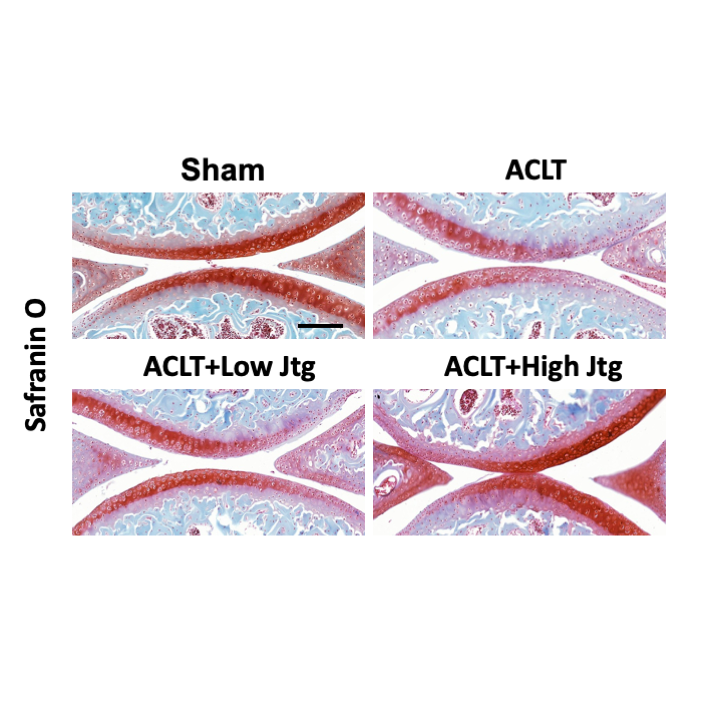
**

**Supplementary Figure 4.** No significant deterioration in cartilage was detected by Safranin O staining at two weeks after the sham or ACLT operation. Scale bar, 200 μm.
